# The Effect of Silica-Filler on Polyurethane Adhesives Based on Renewable Resource for Wood Bonding

**DOI:** 10.3390/polym12102177

**Published:** 2020-09-24

**Authors:** Mariusz Ł. Mamiński, Anna M. Więcław-Midor, Paweł G. Parzuchowski

**Affiliations:** 1 Institute of Wood Sciences and Furniture, Warsaw University of Life Sciences–SGGW, 159 Nowoursynowska St., 02-776 Warsaw, Poland; 2 Faculty of Chemistry, Warsaw University of Technology, 3 Noakowskiego St., 00-664 Warsaw, Poland; awieclaw@ch.pw.edu.pl (A.M.W.-M.); pparzuch@ch.pw.edu.pl (P.G.P.)

**Keywords:** biopolyol, polyurethane adhesive, glycerol, sucrose

## Abstract

The aim of the study was to evaluate the applicability and performance of polyglycerol- and sucrose-based polyols as components of a simplified formulation of polyurethane adhesives. Colloidal silica was used as a viscosity control and reinforcing agent. The adhesives were examined in terms of reactivity, thermal stability, viscosity, work of adhesion, wetting, surface energy, and bonding strength on wooden substrates. Silica was found to increase gelling time, but markedly improved bonding strength and adhesion with substrates. Bonded solid beech wood samples prepared at 80, 110, and 130 °C showed shear strengths between 7.1 MPa and 9.9 MPa with 100% wood failure. The renewable resource-based polyols were demonstrated to be useful in formulation of polyurethane adhesives for furniture industry—especially with silica as a filler.

## 1. Introduction

The research on bio-based or bio-derived adhesives and polyurethanes has been carried out for years [1,2,3,4,5,6,7,8]. Since Anastas and Warner [9] stated twelve principles of green chemistry, the investigations in the field of cleaner production have intensified. One of the most important classes of adhesives is polyurethanes (PURs) that are widely used in wood and furniture industries. Therefore, it is important to increase the content of bio-based components in PUR adhesives—preferably by substitution of petroleum-based polyols with renewable feedstock-derived ones.

The convenient feedstock are glycerol and sucrose. The former is a by-product from vegetable oils transformations to biodiesel [10,11] and the latter is produced in large amounts from sugarcane or sugar beet. Due to their renewability, bio-origin, low cost, and availability, both glycerol and sucrose have great potential as raw material for the development of new classes of polyurethanes [12,13,14]. Incorporation of disaccharides into polyurethane structure results in enhanced hydrolysis in soil which promotes biodegradation [15]. It is also anticipated that the demand on industrial sugar will increase in world markets [16]. Therefore, new ways of industrial use of sucrose should be investigated. The current trends in the adhesives for furniture sector are formaldehyde-free adhesives [17,18], sucrose-based adhesives [19,20], and renewable resource-based polyurethanes [21,22]. Depletion of petroleum supplies, the fluctuating prices and environmental problems which associated with oil industry, make renewable, plant-derived resources more and more attractive for this class of adhesives [23]. Biopolyols are excellent substitutes for petroleum-derived polyols as long as the resultant PURs of high renewable content exhibit properties comparable to traditional ones. Unlike for structural applications, adhesives dedicated to furniture manufacturing—especially for veneering of medium density fiberboards or particleboards—are not required to exhibit very high strength as the surface soundness of these materials typically varies between 0.8 MPa and 1.6 MPa depending on the density of a panel [24,25,26]. No lower limit for the bondline strength in the substrate-adhesive-veneer system is defined. Instead, in practice, a visual analysis of the edge after machining is used for the evaluation of bond quality.

Thus, it was hypothesized that 2-component PUR adhesives of a simplified formulation limited to one biopolyol and one diisocyanate would exhibit properties sufficient for veneering in furniture manufacturing, as well as, decrease the cost of an adhesive. 

A great advantage of polyurethanes is the ease to tailor their properties to a specific area of application such as foams, coatings, or 1- and 2-component adhesives. A formulated adhesive contains a number of components: Isocyanates, a mixture of polyols, catalysts, chain extenders, tackifiers, fillers, and other additives [27,28]. The use of multi-component formulations is to provide an adhesive with high strength, resistance to chemical and physical factors, as well as a long-term durability in service, which is fully justified when adhesive is dedicated to structural applications in construction or engineered wood products. 

In this study two PUR adhesives containing polyglycerol- or sucrose-based polyols were investigated for their application in wood bonding. Industrially feasible adhesives have to meet the requirements such as practical reactivity, viscosity, and bonding strength. These properties have to be tuned for each newly developed binder. The proposed adhesives are expected to be used in veneering in furniture industry. However, the initial experiments indicated that their physical and chemical properties had to be adjusted. The reactivity was adequate, but the low viscosity was a concern due to adhesive overpenetration through a veneer.

It is well studied that silica filler in a polymeric matrix may contribute to increase in mechanical strength and fracture toughness by the mechanism of crack tip blunting or crack pinning [29,30,31,32]. It was shown that doping of polymer composites with nanosilica may be an efficient way to enhancement of their strength and toughness [33]. Silica particles have also been proved to increase adhesion and surface energy of a polyurethane as well as to modify its rheological behavior [27,34]. Improvement of properties like Young’s modulus [35], storage, and loss moduli of a polyurethanes have also been reported [34].

In the present work, the effect of using the colloidal silica as the filler on the reactivity, viscosity, thermal stability, and adhesive interactions were examined and discussed.

## 2. Materials and Methods 

### 2.1. Materials

Two biopolyols were examined as components for PUR wood adhesives: A—polyglycerol synthesized by ring opening polymerization of glycerol carbonate and 1,1,1-tris(hydroxymethyl)-propane (TMP) at 6/1 molar ratio [36,37]. The product was a highly viscous light-brown liquid. NMR spectrum was recorded on a Varian VXR 400 MHz (Palo Alto, CA, USA). ^1^H NMR (400 MHz, DMSO-*d*_6_), *δ* (ppm): 4.91–4.20 (m, 10H, H_2_O, OH), 3.75–3.10 (m, 38H, polyether backbone), 1.36–1.16 (bs, CH_2_CH_3_), 0.77 (bs, CH_2_CH_3_)—see Figure S1. GPC in water: *M*_n_ = 563.47, *M*_w_ = 1036.63, *D* = 1.83 (Viscotek (Malvern Panalytical Ltd., Malvern, UK) system with a triple detector array (TDA) 305 and divinylbenzene (DVB) Jordi column (JordiLabs LLC, Mansfield, MA, USA). It is a structural analogue of the polyglycerol characterized in our previous work [37]. The other commercial sucrose-based polyether polyol labeled as C was manufactured by Oltchim (Valcea, Romania) and used as obtained. Basic properties of the polyols are shown in Table 1. Polymeric methylenediphenyldiisocyanate (PMDI) was purchased from Huntsman Co., (Arlington, TX, USA) and used as received. Isocyanate content in PMDI was 36%, with 4,4′ MDI isomer content lower than 75%. The viscosity of PMDI is 290 mPa·s at 20 °C, and average functionality 2.7. Viscosity of the adhesives was controlled by addition of colloidal hydrophilic silica (SiO_2_, surface areA200 ± 25 m^2^/g manufactured by Evonik Industries AG, Essen, Germany) as a filler: 2.0 wt% for in polyol A and 6.0 wt% for polyol C were needed to reach practical viscosity.

### 2.2. Adhesive Formulation

PMDI and polyols were used in formulation of 2-component PUR adhesives. The amounts of the components were calculated from the Equation (1):

(1) $$m_{iso}=\left(\frac{\sum m_{polyol}\times L_{OH}}{56100}\right)\times \frac{4202}{c_{iso}}$$

where: *m_iso_*—stoichiometric amount of isocyanate in grams, *m_polyol_*—amount of polyol in grams, *L_OH_*—polyol hydroxyl number in mg KOH/g, *c_iso_*—NCO content in PMDI in wt%. Target NCO/OH index was 1.0. The components were weighted in a plastic cup and mixed for 5 s at 500 rpm speed. In the case of SiO_2_-filled polyols, the filler was mixed with a polyol prior to adhesive formulation.

### 2.3. Lap Shear Strength

Bondline shear strength tests were done according to EN 205 [38]. Beech (*Fagus sylvatica*) wood of density 700 ± 50 kg/m^3^ and 5% moisture content was used as the substrate. Adhesives were applied at 200 g/m^2^ rate and bonded at 80, 110, and 130 °C for 7 min under 0.8 MPa pressure.

**Figure 1:**
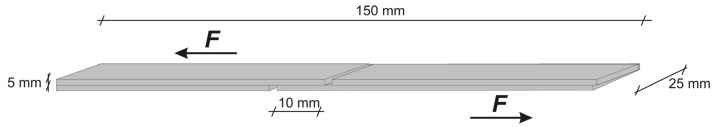
Lap specimen: *F—*force direction.

Bonded specimens (Figure 1) were conditioned at normal conditions (20 ± 2 °C and 65 ± 5% relative humidity) for 7 days before testing. Twelve specimens were tested in each series. 

Shear strength (*R_t_*) was calculated using the Equation (2):

(2) $$R_{t}=\frac{F_{\max}}{S}$$

where: *F*_max_—the maximum force in Newtons, *S*—lap area in mm^2^.

### 2.4. Pull-Off Adhesion Test

**Figure 2:**
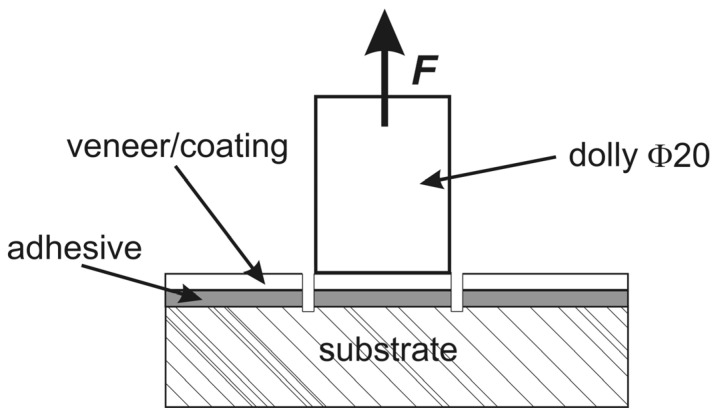
Pull-off specimen: *F—*force direction.

Veneer pull-off tests were performed according to [39] on natural 0.6-mm thick oak veneer bonded to medium density fiberboard (MDF) with the studied adhesives based on polyol A or polyol C (65 °C, 1.0 MPa, 4 min). A Positest^®^AT-A pull-off adhesion tester (DeFelsko Corp., Ogdensburg, NY, USA) equipped with 20-mm dollies was used (Figure 2).

### 2.5. TG-MS Experiments

Thermogravimetric analyses were performed on a STA 449C (Netzsch, Selb, Germany) instrument coupled with QMS 403C (Netzsch, Selb, Germany) mass spectrometer in air (N_2_/O_2_ 79:21, *v*/*v*) at constant heating rate 10 °C/min to 800 °C. Polymer sample of 0.0001 g and 0.25 g calcinated Al_2_O_3_ were placed on a Al_2_O_3_ pan to avoid uncontrolled spill.

MS instrument recorded *m*/*z* of the ions in the range 10–300 Da during thermogravimetric experiments.

### 2.6. Contact Angle (θ), Surface Free Energy (γ), and Work of Adhesion (W_a_)

A Phoenix 300 contact angle analyzer (Surface Electro Optics, SuwonCity, Korea) equipped with microscopic lenses and digital camera was used. The sessile droplet method was used for contact angle and work of adhesion measurements. Distilled water and diiodomethane were used as two reference liquids. The average contact angles are means of 5 measurements done 60 s since droplet deposition. Calculations of surface free energy were based on the Owens–Wendt method [40]. Work of adhesion between wetting liquid (water) and PUR was calculated from the Equation (3) [40]:

*W_a_* = *γ_S_* + *γ_L_* − *γ_SL_* (3)

where: *γ_S_* is the surface energy of solid, *γ_L_* is the surface tension of liquid, *γ_SL_* is the interfacial tension between a solid and a liquid (Equation (4)):

(4) $$\gamma_{SL}=\gamma_{S}+\gamma_{L}-2\sqrt{\gamma_{S}^{D}\gamma_{L}^{D}-2\sqrt{\gamma_{S}^{P}\gamma_{L}^{P}}}$$

where *D* and *P* superscripts refer to dispersive and polar parts of free surface energy. Values for liquids for calculations are presented in Table 2.

## 3. Results and Discussion

### 3.1. Effect of Viscosity on Bonding

**Figure 3:**
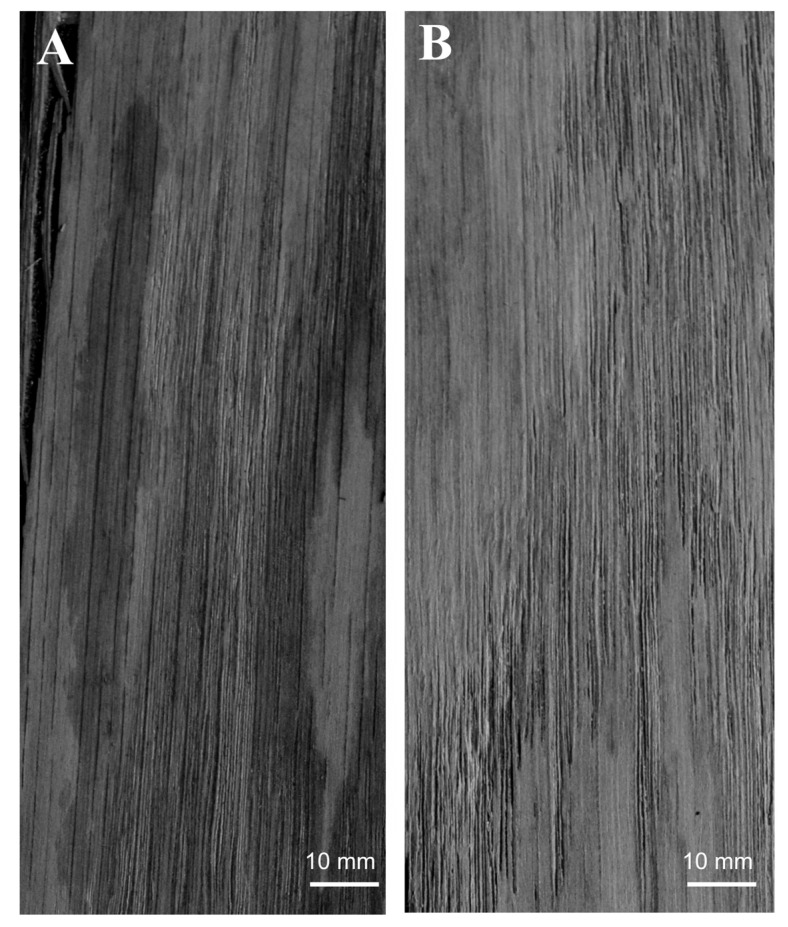
0.6-mm thick oak veneer surface: (**A**) adhesive overpenetration and staining (polyurethane (PUR) without SiO_2_), (**B**) avoided staining (PUR with SiO_2_).

In our initial experiments with PUR adhesives based on glycerol- or sucrose-derived polyols, the viscosities of the formulations without SiO_2_ were too low (1.2 Pa·s and 5.5 Pa·s for C polyol-based and A polyol-based, respectively) which resulted in overpenetration of the adhesive through A0.6-mm thick oak veneer. Thus, surface staining occurred (Figure 3). In order to overcome the problem, the viscosity was adjusted to a practical range by using colloidal silica as the viscosity control agent. It showed that both the addition of 2% silica in A-polyol and 6% silica in C-polyol resulted in a remarkable increase in the viscosities of the polyols (Table 1), while resultant viscosities of the formulated 2-component adhesives were 19.7 Pa·s and 37.0 Pa·s, respectively, for C polyol-based and A polyol-based PURs. Increased viscosity produced thixotropy or pseudoplastic behavior [34], which subsequently, helped to avoid the adhesive overpenetration (Figure 3).

### 3.2. Reactivity

Data in Table 3 indicate the studied adhesive formulations exhibit sufficient reactivity at ambient temperature, as the gelling times between 10 and 15 min at 20 °C is a practical range for solid wood bonding in industry. As it was shown previously, the reactivity of the polyglycerol-based PUR systems can be easily controlled by the type and amount of catalyst used. Thus, set times as short as 1 min can be obtained [37]. However, highly reactive binders are useful only in automatic bonding lines to keep high production capacity—e.g., veneering in furniture industry.

It is worth to mention that, the addition of the SiO_2_ filler decreased the reactivity of PUR. The gelling times at ambient temperature were increased by 17% and 250% for the branched polyglycerol A and the sucrose-based polyol C, respectively. This was caused by the difference amount of silica added, since polyol A was doped with 2%, due to its innate high viscosity, while polyol C was added 6% silica. Apparently, strong H-bonding interactions between polyol molecule and SiO_2_ might have been the main reason of slowing down in the gelling/curing. The observed slowing down of gelling is accordant to the literature data on the thermal effects of PUR curing in the presence of silica. It had been proved that even small additions resulted in a few-fold decrease in enthalpy of curing, thus, the process was hampered and required higher temperature to full conversion [27]. Also, Chiacchiarelli and co-workers demonstrated that nanosilica additions resulted in a decreased the rate constant for curing by ca. 60%, reduced thermal effect and a shift of heat flow peak in DSC curves to higher temperatures [42]. Curing kinetics of the polyglycerol-PMDI systems were described elsewhere [43]. The observed thermal effects depend on the abundancy of hydroxyl groups in a molecule and range between ca. 12 J/g and 50 J/g, while heat flow peak appears about 67–69 °C.

Since veneering usually is performed at the temperature close to 100 °C, high reactivity of the PUR adhesives allows fast processing, and, makes possible to get one-cycle pressing time as short as 30–60 s under industrial conditions.

### 3.3. Bondline Shear Strength 

**Figure 4:**
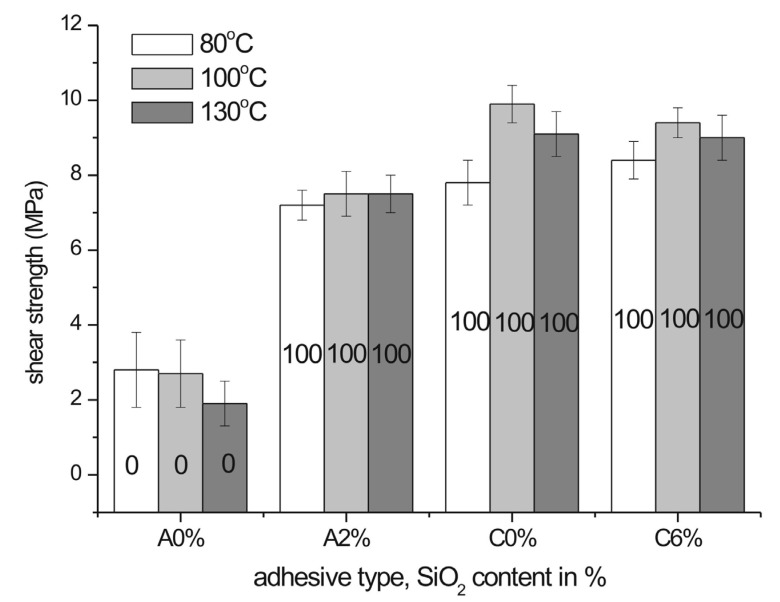
Bondline shear strength of the studied PUR adhesives in solid wood bonded at 80 °C, 110 °C, or 130 °C. Numbers denote percentage of substrate failure (see Figure S2).

It is clear from the analysis of shear strength of the bondlines in solid wood (Figure 4) that pressing temperature does not have remarkable effect on that parameter neither for A nor for C polyol-based adhesive. Thus, bonding can be performed at either temperature without significantly different results. However, due to low strengths of the A polyol-based PUR without SiO_2_ (1.8–2.8 MPa) (Figure 4) its applicability in veneering is discrepant, so that—keeping in mind that the practical surface soundness of particleboard ranging from 0.8 to 1.6 MPa [26,39,44]—evaluation in pull-off test is necessary. Comparable shear strengths (2.84 MPa with high rate of substrate failure were reported by Tenorio-Alfonso and co-workers [22] for the adhesives derived from cellulose acetate, 1,6-hexamethylene diisocyanate and castor oil with renewable content as high as 70%. Similar results were achieved for hot melt reactive polyurethanes derived from CO_2_-based poly(propylene carbonate): Lap-shear tests performed after 7-day curing on aluminum and steel revealed maximum strengths, respectively, 5.5 MPa and 4.7 MPa [45] and 1.5–6.0 MPa for plastics [46]. PUR adhesives based on biopolyols from liquefied biomass were proved to exhibit comparable performance as the shear strengths ranged between 5.8 and 11.0 MPa [21].

On the other hand, addition of silica had more apparent effect on bonding strength. The comparison of the values for non-SiO_2_-filled and SiO_2_-filled adhesive formulations revealed significant increase in shear strength, but the effect was more evident for A polyol-based adhesive of low innate strength: 1.8–2.8 MPa and 7.2–7.5 MPa for non-filled and filled, respectively. A lower performance of the A polyol-based adhesive without SiO_2_ (A0% series) can be attributed to dense crosslinking due to A polyol hydroxyl functionality as high as 9 which renders brittleness and low mechanical properties. Dispersed silica presence resulted in improving of the mechanical properties of the adhesive (A2% series) according to the mechanisms reported in [29,32]. Unlike the A-series, the C polyol-based adhesive in shear strength tests was not apparently affected by silica addition. The used wood substrate—though conforming the standards—did not allow measure true strength of the adhesive. The observed 1.7- to 3-fold enhancement as well as high substrate failure rate indicate that the approach was more effective than that reported for thermoplastic PURs composites [34] or nanosilica-filled PUR adhesive where 8% gain was achieved [27] and coherent with the results obtained for acrylic-polyurethane coatings doped with 4–6% nanosilica where 40% enhancement in adhesion was yielded [47]. Worth mentioning is that the performance of the SiO_2_-filled PURs is comparable to that of the commercial adhesives not containing bio-derived components—i.e., PUR 6.25 MPa (100% substrate failure) [48]. The phenomenon can be explained: (1) by the development of the hydrogen bonds between the hydroxyl groups of the nanoparticles and the carbonyl groups of the polyurethane [49] which intensifies interfacial forces and is accordant with the chemical bonding theory of adhesion and (2) by the role of the filler in fracture formation. According to the crack pinning mechanism, rigid particles impede crack propagating, so that fracture toughness of a filled adhesive is increased [30,31]. The effect was not manifested for the polymer of high innate strength—i.e., PUR based on polyol C (Figure 4). Strengthening effect of small additions of silica on the mechanical properties of polyurethanes remains in accordance with the results reported in recent literature [50,51]. 

### 3.4. Wetting and Surface Properties

**Figure 5:**
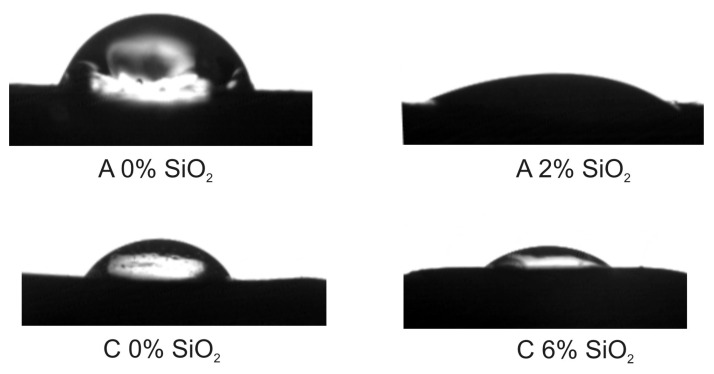
Water contact angle for non-SiO_2_-filled and SiO_2_-filled PURs 60 s after droplet deposition.

As the analyses of contact angle, surface free energy and work of adhesion indicate the addition of SiO_2_ resulted in a lower water contact angle (Figure 5) and increased work of adhesion (Table 4). A 17.9% increase in *W*_a_ was observed for the C-based PUR and 23.6% for the A-based PUR. In this work, the contact angles for water due to silica addition were 23°–27°, while that observed for nanosilica-filled PURs reported in [27] was 53°–67° as well as surface free energy enhanced 44% which is 2-times that found in the literature for PURs with nanosilica doped at a comparable level (4.1 wt%).

Work of adhesion values are slightly higher to those reported for water-beech or water-oak wood interactions (126–133 mJ/m^2^) [40]. As interactions between PUR and water are enhanced and water is considered the model of a polar phase, it can be inferred that PUR interactions with hydrophilic wood surface containing high concentration of OH groups (ca. 10^21^ groups per gram [52]) are also enhanced. The phenomenon confirms that filling the adhesive with SiO_2_ was beneficial for adhesion between veneer and adhesive and the observed changes in surface properties promote adhesive interactions. The significance of substrate/adhesive surface energy relation on the adhesion forces has been recognized and associated with interfacial tension [40,53]. As Equation (3) indicates *W_a_* is maximized when the interfacial tension *γ_SL_* is equal zero. Thus, as silica addition induced an increase in PUR surface energy, the interfacial tension *γ_SL_* was lowered and, subsequently, work of adhesion increased, which resulted in increased PUR/substrate interactions.

### 3.5. Pull-Off Adhesion Test

**Figure 6:**
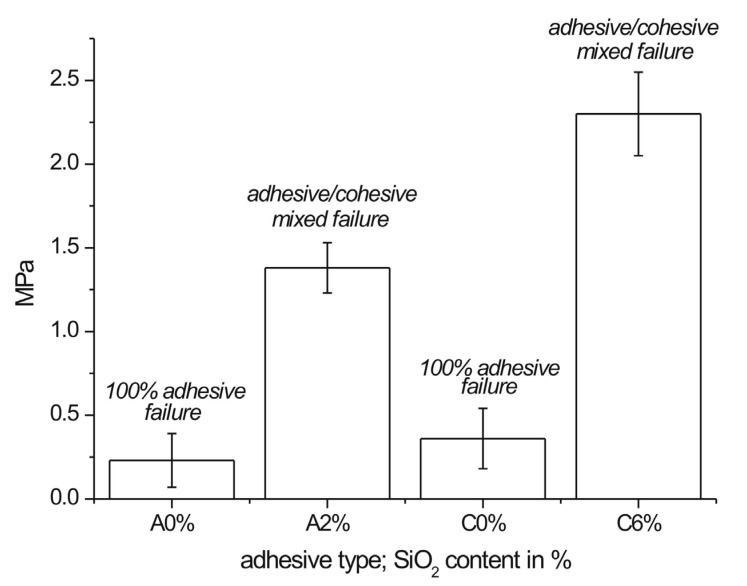
Bondline strength in pull-off tests performed on medium density fiberboard (MDF) veneered with 0.6-mm thick oak veneer.

In order to assess the applicability of adhesives in veneering, a good bond in pull-off test is even more important than that in the lap shear strength. It showed that a significant increase in bonding between a veneer and a substrate can be induced by silica addition. The effect is apparent as the investigated adhesives that exhibit innate low strengths. It is worth to mention that the adhesives for veneering are not required to render substrate failure (MDF/particleboard). It is desirable, but not required by any European standard. The bonding strengths of both investigated PURs (A2% vs. A0% and C6% vs. C0%) are shown in Figure 6. The revealed improvement in pull-off adhesion tests remains in accordance with the increased work of adhesion and surface free energy of the adhesive with SiO_2_ (Table 4). Thus, the enhanced bonding between wooden materials and the silica-filled PURs has been clearly demonstrated. The values shown in Figure 6 are comparable or slightly lower to those achieved for commercial non-renewable-based PUR (2.50 MPa) [22].

One can notice a poor performance of the C0% PUR in the pull-off test (Figure 6), while the strengths observed in the lap shear tests were satisfactory (100% substrate failure) (Figure 4). A possible explanation can low viscosity (1.2 Pa·s at 25 °C) of the adhesive, which, in contact with porous substrates (MDF) and oak veneer, intensely penetrated both materials. Subsequently, it is likely that too thin bondline was formed. Unlike for MDF substrate, C0% PUR penetration into less porous solid wood in lap shear specimens was weaker, which resulted in an improved bondline performance. Viscosity increase to 19.7 Pa·s due to silica addition impeded overpenetration of the adhesive into substrates.

### 3.6. Thermal Properties

Thermogravimetric experiments revealed (Table 5, Figure S3) that the presence of silica affects thermal degradation of the polymers. In all cases temperatures of a indicated weight loss were shifted to a lower value. It is surprising as the increase in PUR thermal stability in the presence of silica has been recognized in the literature [54,55]. 

**Figure 7:**
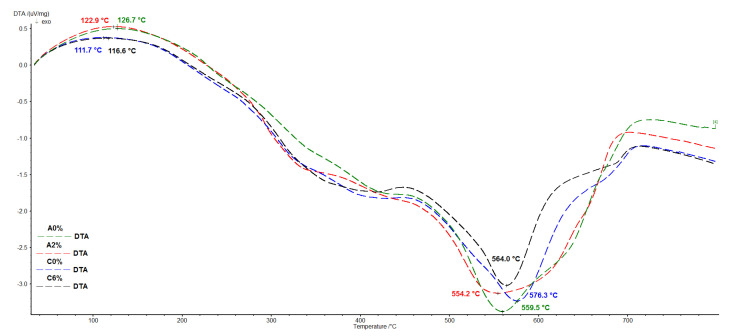
DTA curves for the investigated PURs.

DTA curves shown in Figure 7 indicate that the endothermic peaks in region 110–125 °C correspond to dehydration of polymers while exothermic peaks around 554.2–576.3 °C are associated with thermooxidation. MS results coupled with TG presented in Figure S3 proved that the main thermooxidation products are water (*m*/*z* 18) and CO_2_ (*m*/*z* 44). Maximum water evolution was found at 390.2 °C and 400.5 °C for the A0% SiO_2_ and A2% SiO_2_ PURs, respectively, and at 380.3 °C and 359.2 °C for the C0% SiO_2_ and C6% SiO_2_ PURs. Maxima for carbon dioxide evolution were observed at 597.4 °C for C0% SiO_2_ PUR and at 566.9 °C for C6% SiO_2_ PUR.

From the practical point of view the changes in thermal stability of the PURs can be neglected as in the area of their target application high thermal resistance is not required.

## 4. Conclusions

The presented results clearly demonstrate that polyglycerol- and sucrose-based polyols can be considered renewable resource-based materials for 2-component polyurethane adhesive formulations. It has been shown that the properties of the adhesives (reactivity and bonding strength) of a formulation simplified to one polyol can be easily tuned by silica so that become applicable in industrial veneering operations. 

The observations also revealed that small additions of colloidal silica used as a viscosity control agent decreased PURs reactivity and thermal stability, but on the other hand, improved adhesion interactions with the substrate, reinforced polymers, and increased bondline strengths. 

Therefore, the polyglycerol- and sucrose-based polyols seem useful raw materials for further development and optimization of adhesives to be implemented in furniture industry.

## Figures and Tables

**Table 1:** Selected physical and chemical properties of the studied polyols.

Polyol	Hydroxyl Number [mg KOH/g]	Hydroxyl Functionality	Viscosity at 25 °C [Pa·s]	
**-**	-	-	Non-filled	SiO_2_-filled
A	694	9 ^a^	10	400
C	425	5	0.70	275

^a^ based on theoretical structure; A—polyglycerol polyol; C—commercial sucrose-base polyol.

**Table 2:** Surface tension components of the reference liquids (mJ/m^2^) [41].

Liquid	γ	γ^D^	γ^P^
Diiodomethane	50.80	50.80	0.00
Water	72.80	21.90	51.00

**Table 3:** Gelling time at 20 °C for polyglycerol- and sucrose-based PUR adhesives (NCO/OH ratio 1.0).

Polyol	SiO_2_-Filler Addition [wt%]	Gelling Time [s]
A	0	510
A	2.0	600
C	0	600
C	6.0	2100

**Table 4:** Wetting, surface energy and work of adhesion of the studied PURs.

Characteristics	-	Polyol Used			
	-	A0% SiO_2_	A2% SiO_2_	C0% SiO_2_	C6% SiO_2_
contact angle θ [°]	water	56.9 ± 2.3	23.1 ± 4.0	53.5 ± 1.6	27.7 ± 0.7
	diiodomethane	45.3 ± 2.4	17.8 ± 0.3	38.1 ± 0.9	38.0 ± 2.2
surface free energy [mJ/m^2^]		51.7 ± 2.0	75.0 ± 1.3	55.0 ± 1.7	70.7 ± 1.8
work of adhesion [mJ/m^2^]		112.0 ± 1.5	139.7 ± 1.8	116.5 ± 1.6	137.4 ± 2.0

**Table 5:** Thermal stability of the studied PURs.

Sample	Temperature of Weight Loss [°C]			Residual Weight at 800 °C [%]
	5%	50%	80%	
A0% SiO_2_	207.8	421.2	592.4	1.21
A2% SiO_2_	189.0	406.1	580.4	3.87
C0% SiO_2_	163.3	387.3	567.3	1.20
C6% SiO_2_	156.2	367.7	559.5	6.25

## Supplementary Materials

The following are available online at https://www.mdpi.com/2073-4360/12/10/2177/s1, Figure S1: NMR and FTIR spectra of the polyglycerol; Figure S2: Representative adhesive and substrate failure; Figure S3: TG and MS curves of the studied PURs.
