# Supplementary material for: The Effect of Silica-Filler on Polyurethane Adhesives Based on Renewable Resource for Wood Bonding
Source: Polymers (Basel). 2020 Sep 24;12(10):2177. doi: 10.3390/polym12102177 (PMC7598712; doi:10.3390/polym12102177)
Supplement: Supplementary file 1 [file polymers-12-02177-s001.pdf]

Supplementary Materials

The Effect of Silica-Filler on Polyurethane Adhesives Based on Renewable Resource for Wood Bonding

Mariusz Ł. Mamiński [1,*], Anna M. Więcław-Midor, [2] and Paweł G. Parzuchowski [2]

[1] Institute of Wood Sciences and Furniture, Warsaw University of Life Sciences–SGGW, 159 Nowoursynowska St., 02-776 Warsaw, Poland

[2] Faculty of Chemistry, Warsaw University of Technology, 3 Noakowskiego St., 00-664 Warsaw, Poland; awieclaw@ch.pw.edu.pl (A.M.W.-M.); pparzuch@ch.pw.edu.pl (P.G.P.)

* Correspondence: mariusz_maminski@sggw.edu.pl; Tel.: +48-22-593-85-27

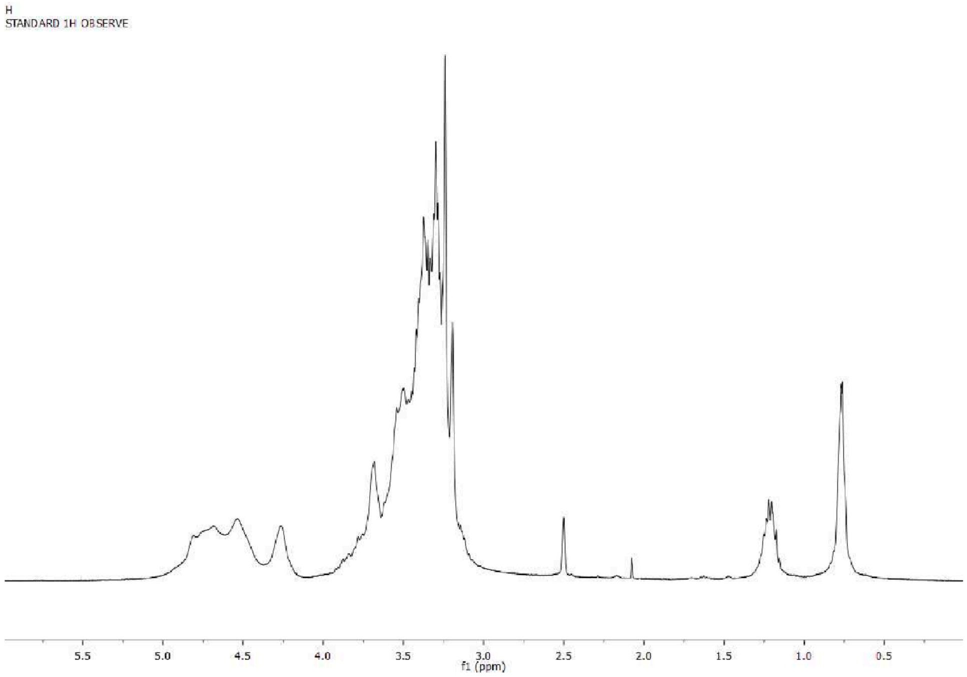

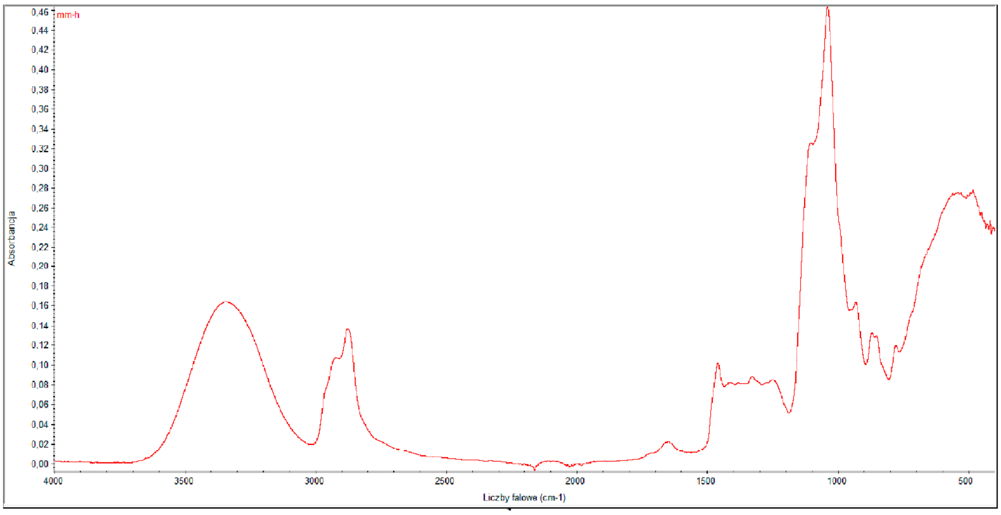

Figure S1. 1H NMR of the studied polyglycerol: (400 MHz, D2O), δ (ppm): 4.80-3.90 (8H, H2O, OH), 3.75–3.10 (34H, polyether backbone), 1.25 (CH2CH3), 0.75 (CH2CH3) (upper image); FTIR of the studied polyglycerol: (film); cm-1 = 3350 (OH), 2900 and 2870 (CH & CH2), 1070 (–O-C-O–) (lower image).

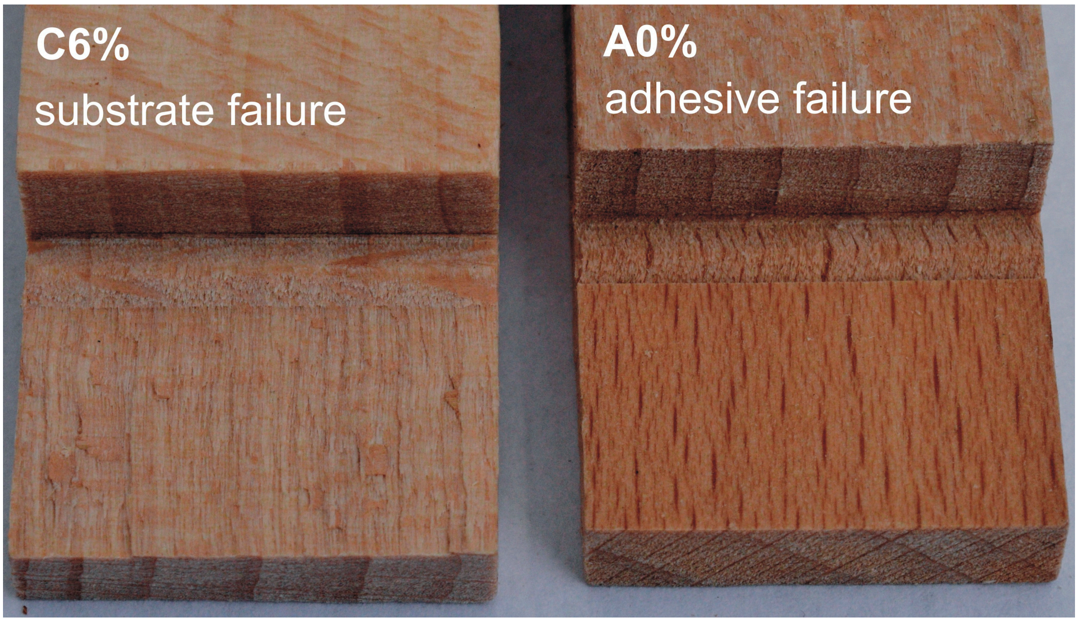

Figure 2. Representative adhesive and substrate failure.

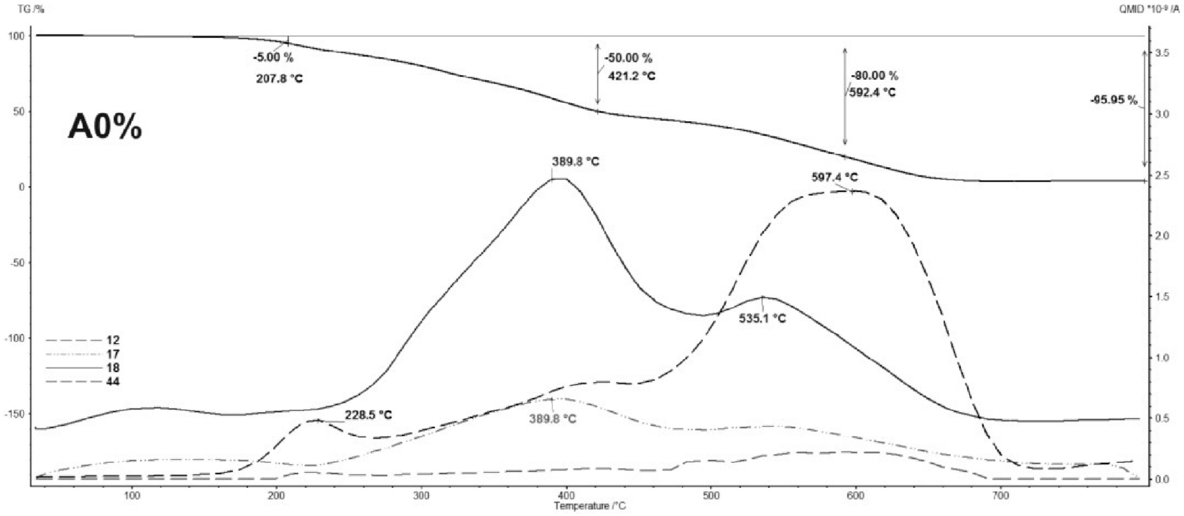

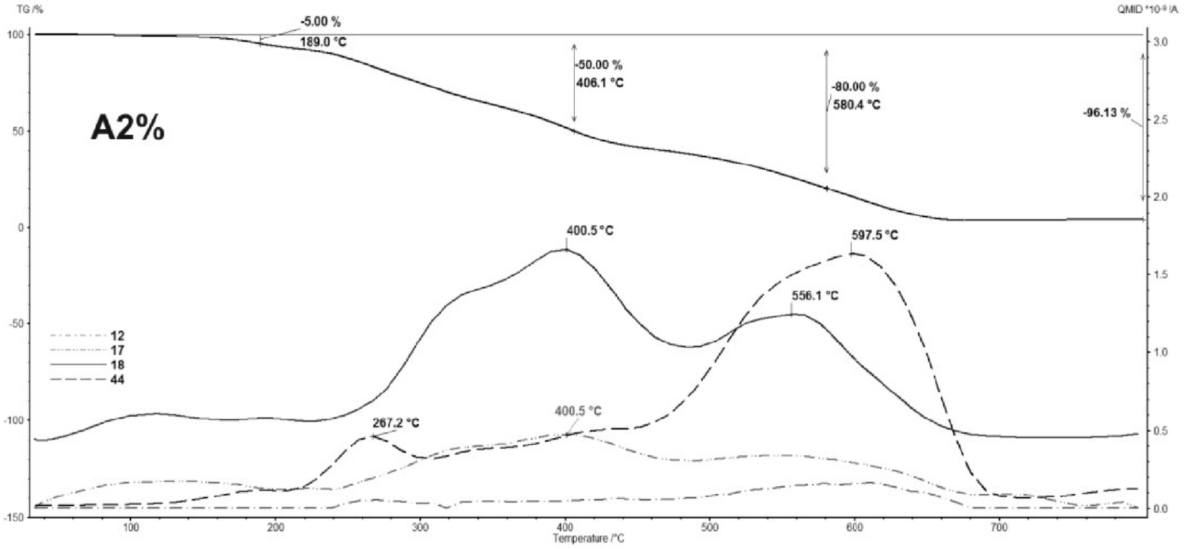

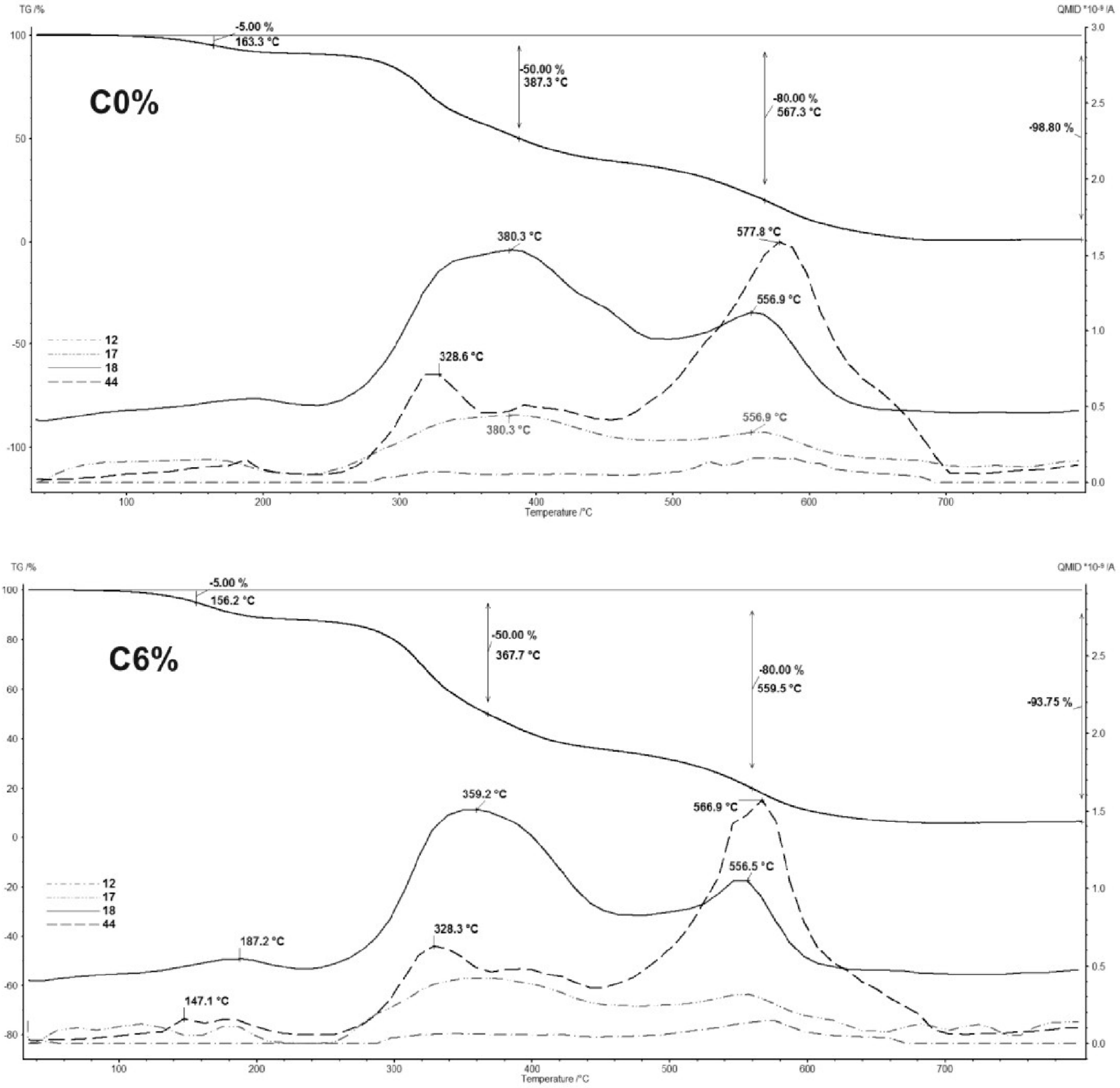

Figure 3. TG-coulped MS curves of the studied PURs.
